# Clinical and computed tomographic predictors of chronic bronchitis in COPD: a cross sectional analysis of the COPDGene study

**DOI:** 10.1186/1465-9921-15-52

**Published:** 2014-04-27

**Authors:** Victor Kim, Adam Davey, Alejandro P Comellas, Meilan K Han, George Washko, Carlos H Martinez, David Lynch, Jin Hwa Lee, Edwin K Silverman, James D Crapo, Barry J Make, Gerard J Criner

**Affiliations:** 1 Temple University School of Medicine, 785 Parkinson Pavilion, 3401 North Broad Street, Philadelphia, Pennsylvania 19140, USA; 2 Department of Public Health, Temple University, Philadelphia, PA, USA; 3 University of Iowa Hospital and Clinics, Iowa City, IA, USA; 4 University of Michigan, Ann Arbor, MI, USA; 5 Brigham and Women’s Hospital, Boston, MA, USA; 6 Ewha Womans University, Seoul, Korea; 7 National Jewish Health, Denver, CO, USA

**Keywords:** Chronic bronchitis, Chronic obstructive pulmonary disease, Airway thickening, Asthma

## Abstract

**Background:**

Chronic bronchitis (CB) has been related to poor outcomes in Chronic Obstructive Pulmonary Disease (COPD). From a clinical standpoint, we have shown that subjects with CB in a group with moderate to severe airflow obstruction were younger, more likely to be current smokers, male, Caucasian, had worse health related quality of life, more dyspnea, and increased exacerbation history compared to those without CB. We sought to further refine our clinical characterization of chronic bronchitics in a larger cohort and analyze the CT correlates of CB in COPD subjects. We hypothesized that COPD patients with CB would have thicker airways and a greater history of smoking, acute bronchitis, allergic rhinitis, and occupational exposures compared to those without CB.

**Methods:**

We divided 2703 GOLD 1–4 subjects in the Genetic Epidemiology of COPD (COPDGene®) Study into two groups based on symptoms: chronic bronchitis (CB+, n = 663, 24.5%) and no chronic bronchitis (CB-, n = 2040, 75.5%). Subjects underwent extensive clinical characterization, and quantitative CT analysis to calculate mean wall area percent (WA%) of 6 segmental airways was performed using VIDA PW2 (http://www.vidadiagnostics.com). Square roots of the wall areas of bronchi with internal perimeters 10 mm and 15 mm (Pi10 and Pi15, respectively), % emphysema, %gas trapping, were calculated using 3D Slicer (http://www.slicer.org).

**Results:**

There were no differences in % emphysema (11.4 ± 12.0 vs. 12.0 ± 12.6%, p = 0.347) or % gas trapping (35.3 ± 21.2 vs. 36.3 ± 20.6%, p = 0.272) between groups. Mean segmental WA% (63.0 ± 3.2 vs. 62.0 ± 3.1%, p < 0.0001), Pi10 (3.72 ± 0.15 vs. 3.69 ± 0.14 mm, p < 0.0001), and Pi15 (5.24 ± 0.22 vs. 5.17 ± 0.20, p < 0.0001) were greater in the CB + group. Greater percentages of gastroesophageal reflux, allergic rhinitis, histories of asthma and acute bronchitis, exposures to dusts and occupational exposures, and current smokers were seen in the CB + group. In multivariate binomial logistic regression, male gender, Caucasian race, a lower FEV_1_%, allergic rhinitis, history of acute bronchitis, current smoking, and increased airway wall thickness increased odds for having CB.

**Conclusions:**

Histories of asthma, allergic rhinitis, acute bronchitis, current smoking, a lower FEV_1_%, Caucasian race, male gender, and increased airway wall thickness are associated with CB. These data provide clinical and radiologic correlations to the clinical phenotype of CB.

## Introduction

Chronic bronchitis (CB) affects 18-45% of COPD subjects and has been linked to multiple clinical sequelae including an increased exacerbation rate, accelerated decline in lung function, worse health-related quality of life (HRQoL), and possibly increased mortality [1-3]. The pathologic correlate to CB is goblet cell hyperplasia, which is well described in both the large and small airways in COPD subjects [4,5]. Small airway mucus burden has been linked to mortality and changes in lung function after lung volume reduction surgery [6,7]. A large lung pathology study also found that the number of small airways occluded by mucus plugs increased as COPD disease severity worsened [8].

Despite the clinical importance of CB on outcomes, our understanding of its pathophysiology is surprisingly poor. It is unclear why some individuals develop chronic bronchitis whereas others do not, despite similar environmental exposures. A thirty year longitudinal study found that 42% of continued smokers, 26% of ex-smokers, and 22% of never smokers developed CB [9]. To complicate matters, the correlation between symptoms and goblet cell hyperplasia is poor, and the most well described pathologic index of CB is weak at best [10]. An analysis of the National Emphysema Treatment Trial found no relationship with the severity of cough and sputum with small airway occlusion by mucus [11]. Thus, a better understanding of chronic bronchitis from a pathologic standpoint is needed.

An emerging surrogate for pathologic study is CT quantitative analysis of airway dimensions and emphysema. From a clinical standpoint, we have shown that chronic bronchitics in a group of 1,061 subjects from the COPDGene study with moderate to severe airflow obstruction were younger, more likely to be current smokers, male, Caucasian, had worse health related quality of life, more dyspnea, and increased exacerbation history compared to those without CB [2]. We sought to further refine our clinical characterization of chronic bronchitics in a larger cohort, analyze the CT correlates of CB in COPD subjects, and develop a prediction model for CB with these characteristics. We hypothesized that subjects with CB would have thicker airway walls, less emphysema, a greater percentage of current smoking, a greater overall history of smoking, a greater exposure to environmental fumes and dusts, and more allergic upper airway symptoms.

## Methods

### Patient selection

The Genetic Epidemiology of COPD (COPDGene®) Study is a multicenter observational study that recruited over 10,000 subjects to analyze genetic susceptibility for the development of COPD. This study underwent IRB approval at all centers. Inclusion and exclusion criteria and protocol were described previously [12]. Briefly, enrollees are African-American or non-Hispanic Caucasian between the ages of 45 and 80 with at least a 10 pack-year smoking history. Exclusion criteria include pregnancy, history of other lung disease except asthma, prior lobectomy or lung volume reduction, active cancer undergoing treatment, or known or suspected lung cancer. We used the March 2013 dataset with 10,192 subjects and included those with GOLD 1 through 4 COPD that had complete medical histories and quantitative CT assessments in our analysis. Subjects from our prior analysis [2] were included in this study.

**Figure 1:**
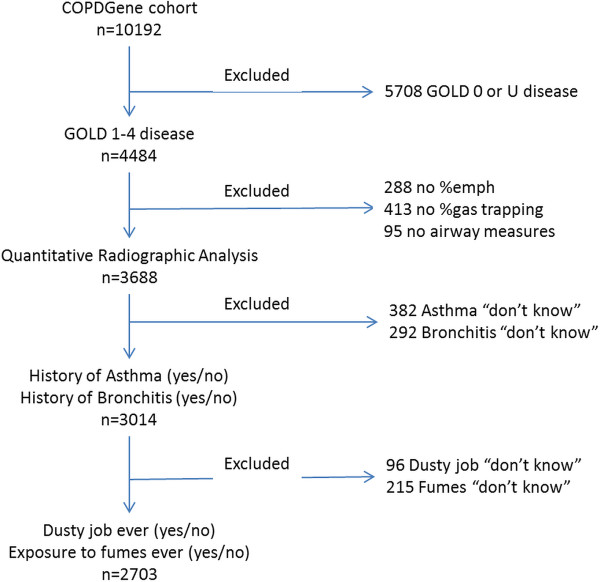
Selection of cohort.

Subjects were asked if they had cough; if they responded yes, they were asked if they coughed on most days for 3 consecutive months or more each year and for how many years. Similar questions were asked regarding phlegm production. Subjects were placed in 1) the chronic bronchitis group (CB+), if they had chronic cough and phlegm production for 3 months or more each year for at least two consecutive years; or 2) the no chronic bronchitis group (CB-), if these criteria were not satisfied. Figure 1 demonstrates the patient selection process.

### Clinical characterization

Upper and lower respiratory tract symptoms were collected using a modified form of the American Thoracic Society Division of Lung Disease (ATS-DLD) Respiratory Epidemiology questionnaire. Medical comorbidities and occupational exposures were assessed based on subject self-report. Of particular interest were histories of asthma, episodes of acute bronchitis, allergic ocular and nasal symptoms, exposures to gases and fumes (defined as occupational exposures), exposures to dusts, and gastroesophageal reflux disease. The definition of acute bronchitis was determined by the answer to the question “Have you ever had an attack of bronchitis?” Subjects were asked specifically if they had these conditions and were instructed to answer yes or no. Subjects that answered “don’t know” to these questions were excluded from the analysis. Subjects were also asked if they experienced COPD exacerbations in the past year and to quantify the number of episodes, and if they have been to the emergency room or hospitalized for an exacerbation in the past year (defined as severe exacerbations). An exacerbation was defined as a flare-up of chest trouble requiring treatment with antibiotics and/or steroids. These answers were used to determine exacerbation history and history of severe exacerbations, respectively. Each subject underwent pre- and post-bronchodilator spirometry using an EasyOne™ spirometer (Zurich, Switzerland). Predicted values were obtained using NHANES III data. Six minute walk distance was measured in standard fashion [13].

### Computed tomography

Volumetric chest CT acquisitions were obtained at full inspiration (200 mAs), and at the end of normal expiration (50 mAs) [12]. Thin-slice collimation with slice thickness and intervals of < lmm was used to enhance spatial resolution. Quantitative image analysis to calculate percent emphysema and percent gas-trapping was performed using 3D SLICER (http://www.slicer.org/). Percent emphysema was defined as the total percentage of both lungs with attenuation values less than −950 Hounsfield units on inspiratory images, and percent gas trapping was defined as the total percentage of both lungs with attenuation values less than −856 Hounsfield units on expiratory images. Airway disease was quantified as wall area percent (WA%: (wall area/total bronchial area)x100) using VIDA (http://www.vidadiagnostics.com) [14]. The mean WA% were calculated as the average of the values for six segmental bronchi in each subject. Using 3D SLICER, airway wall thickness was also expressed as the square root of the wall area of a hypothetical 10 mm and 15 mm internal perimeter airway (Pi10 and Pi15, respectively) as previously described [15].

### Statistical analysis

Statistical analysis was performed using Stata 13. Values are expressed as mean ± SD unless otherwise stated. CB + was the outcome of interest. The covariates analyzed in the model included gender, race, current smoking, smoking history, gastroesophageal reflux, COPD severity based on FEV_1_ and FVC, allergic eye and nasal symptoms, occupational exposures, exposure to dusts, history of asthma, history of acute bronchitis, and quantitative CT abnormalities (Pi10, Pi15, WA% seg). To reduce multicollinearity, principal axis factoring was used to estimate a wall thickness composite. Multivariable logistic regression using a trimmed covariate set selected via backward elimination with robust standard errors adjusting for within-clinic clustering was used to predict CB with all covariates associated with CB at p < .1 in at least one model retained. Interactions between predictors of interest and sex and race were considered in sensitivity analyses. All relevant assumptions were evaluated in the usual fashion (e.g., linearity of association, link tests, hat tests).

## Results

The patient characteristics and univariate analysis of the groups are summarized in Table 1. There were a total of 4484 GOLD 1–4 subjects; of those with complete medical history and quantitative CT analysis, there were 2703 subjects, 2040 subjects in the CB- group and 663 in the CB + group (24.5% of total). The CB + group was younger, had a greater smoking history, and was more likely to be current smokers, Caucasian, and male. The CB + group had a lower forced expiratory volume in 1 second (FEV_1_), forced vital capacity (FVC) and FEV_1_/FVC ratio. 6-minute walk distance was lower and MMRC dyspnea and BODE scores were higher in the CB + group as well. More subjects in the CB + group described gastroesophageal reflux (relative risk [RR] 1.2), allergic nasal and ocular symptoms (RRs 1.5 and 1.3, respectively), a history of asthma and acute bronchitis (RRs 1.4 and 1.3, respectively), and were more likely to be exposed to dusts and occupational exposures (RRs 1.4 and 1.3, respectively). Total exacerbation rate in the year prior to enrollment and history of severe exacerbations were higher in the CB + group as well. In a multivariable linear regression with FEV_1_, FVC, race, gender, mMRC dyspnea score, BMI, and CB as predictors of interest, CB was a significant predictor exacerbation frequency (β coefficient 0.295, p < 0.0001). The CB + group had a greater mean segmental wall area percent, Pi10, and Pi15. There were no differences in quantitative measures of percent emphysema or percent gas trapping between groups.

**Table 1:** Baseline characteristics and radiology

	**Chronic bronchitis (CB+) n = 663**	**No chronic bronchitis (CB-) n = 2040**	**p**
*Demographics and symptoms*			
Age (years)	61.8 ± 8.5	63.8 ± 8.5	**<0.0001**
Smoking history (pack years)	56.4 ± 26.8	50.7 ± 26.3	**<0.0001**
Current smokers (%)	59.2	37.4	**<0.0001**
Gender (%M)	64	55	**<0.0001**
Race (%C)	84	78	**<0.0001**
FEV_1_ (% predicted)	54.3 ± 20.7	60.1 ± 23.4	**<0.0001**
FVC (% predicted)	80.5 ± 19.9	84.0 ± 20.1	**<0.0001**
FEV_1_/FVC	0.51 ± 0.13	0.53 ± 0.14	**<0.0001**
6MWD (meters)	372 ± 118	392 ± 121	**<0.0001**
BMI (kg/m^2^)	27.3 ± 5.9	27.7 ± 5.8	0.144
BODE score	3.0 ± 2.0	2.3 ± 2.1	**<0.0001**
MMRC dyspnea	2.3 ± 1.4	1.6 ± 1.5	**<0.0001**
SGRQ score	48.0 ± 21.3	30.6 ± 21.8	**<0.0001**
*Exposures and Medical History*			
Gastroesophageal reflux (%)	33.8	28.6	**0.010**
Allergic nasal symptoms (%)	69.5	47.0	**<0.0001**
Allergic ocular symptoms (%)	51.0	37.9	**<0.0001**
History of asthma (%)	33.4	23.4	**<0.0001**
History of bronchitis (%)	66.0	49.8	**<0.0001**
Exposure to dusts (%)	64.7	47.4	**<0.0001**
Occupational exposures (%)	65.6	51.0	**<0.0001**
*Exacerbations*			
Total exac rate (no./pt/yr)	0.96 ± 1.46	0.52 ± 1.04	**<0.0001**
History of severe Exac (%)	24.2	15.2	**<0.0001**
*Radiology*			
% Emphysema (%)	11.4 ± 12.0	12.0 ± 12.6	0.347
% Gas trapping (%)	35.3 ± 21.2	36.3 ± 20.6	0.272
Mean segmental WA% (%)	63.0 ± 3.2	62.0 ± 3.1	**<0.0001**
Pi10 (mm)	3.72 ± 0.15	3.69 ± 0.14	**<0.0001**
Pi15 (mm)	5.24 ± 0.22	5.17 ± 0.20	**<0.0001**

Definition of abbreviations: M = male, F = female, C = Caucasian, AA = African American, FEV1 = forced expiratory volume in 1 second, FVC = forced vital capacity, 6MWD = 6-minute walk distance, Exac = exacerbation, BMI = body mass index.

To assess for the possible confounding effects of active smoking, we performed a separate univariate analysis of active smokers versus those that did not currently smoke on the same variables. Active smokers walked a greater distance in 6 minutes (1288.58 ± 390.69 vs. 1189.95 ± 415.72 feet, p < 0.0001), were less dyspneic (MMRC scores 1.69 ± 1.48 vs. 2.07 ± 1.43, p < 0.0001), had lower SGRQ scores (36.07 ± 23.79 vs. 37.50 ± 22.22, p = 0.038), had better lung function (FEV_1_ 63.5 ± 21.3 vs. 52.7 ± 22.8%, FVC 85.9 ± 19.9 vs. 78.8 ± 20.2%. p < 0.0001 for both), and had less emphysema and gas trapping (6.79 ± 8.83 vs. 15.25 ± 13.07% and 28.03 ± 18.88 vs. 41.58 ± 20.35%, respectively, p < 0.0001 for both). Fewer active smokers described allergic ocular or nasal symptoms, gastroesophageal reflux, or severe exacerbations. Airway wall thickness, however, was greater in active smokers, as measured by Pi10, Pi15, and WA% segmental.

### Principal axis factoring

Principal axis factoring was used to reduce collinearity in wall area thickness measures. Two factors accounted for essentially 100% of the shared variance among wall area thickness measures, and each item loaded highly on only one factor. The rotated pattern matrix is shown in Table 2. Quartimax rotated factor scores were constructed using the regression method and agreed well (r > .99) with principal components results.

**Table 2:** Quartimax rotated factor pattern matrix

**Variable**	**WA thickness**	**Emphysema**	**Uniqueness**
ln % Emph	−0.1199	0.8883	0.1965
% Gas trap	0.0561	0.8916	0.2019
Pi10	0.6983	0.0327	0.5114
Pi15	0.8393	−0.1173	0.2819
WA% seg	0.8533	0.0224	0.2714

**Figure 2:**
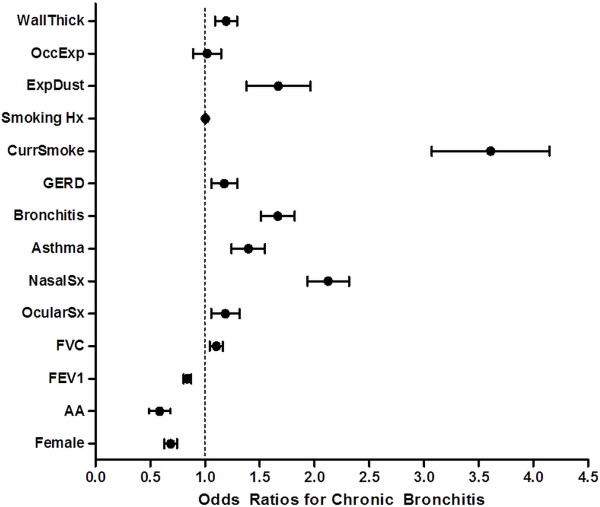
Odds ratios for chronic bronchitis.

Table 3 summarizes the multivariate binomial logistic regression of the predictors of interest with chronic bronchitis as the outcome. In this multivariate analysis, there was no independent association between smoking history or occupational exposures and chronic bronchitis. Female gender and African American race were associated with lower odds ratios of CB (OR 0.68, 95% CI 0.59-0.79, and OR 0.57, 95% CI 0.43-0.76, respectively). Lower FEV_1_ and higher FVC were associated with a greater risk of CB (ORs 0.84, 95% CI 0.78-0.90 and 1.10, 95% CI 1.00-1.21, respectively, per 10% increase). The ORs for allergic ocular symptoms, allergic nasal symptoms, history of asthma, history of acute bronchitis, gastroesophageal reflux, and current smoking were 1.17, 2.11, 1.38, 1.65, 1.16, and 3.53, respectively (see Table 3 for 95% CIs). A composite value of the three CT airway variables (called wall thickness) conferred an OR of 1.19 (95% CI 1.02-1.37) for chronic bronchitis. Figure 2 shows the odds ratios for the predictors of interest.

**Table 3:** Odds ratios for chronic bronchitis in a multivariate logistic regression model

	**OR**	**95% CI**		**p**
Wall thickness	1.19	1.02	1.37	0.022
Female gender	0.68	0.59	0.79	<0.0001
African American race	0.57	0.43	0.76	<0.0001
FEV_1_ (per 10% predicted)	0.84	0.78	0.90	<0.0001
FVC (per 10% predicted)	1.10	1.00	1.21	0.043
Allergic ocular symptoms	1.17	0.97	1.42	0.105
Allergic nasal symptoms	2.11	1.81	2.46	<0.0001
History of asthma	1.38	1.14	1.67	0.001
History of bronchitis	1.65	1.41	1.94	<0.0001
Gastroesophageal reflux	1.16	0.98	1.39	0.092
Current smoking	3.53	2.72	4.58	<0.0001
Smoking history (pack years)	1.004	1.003	1.007	0.035
Exposure to dusts	1.62	1.19	2.20	0.002
Occupational exposures	1.00	0.81	1.25	0.996

**Figure 3:**
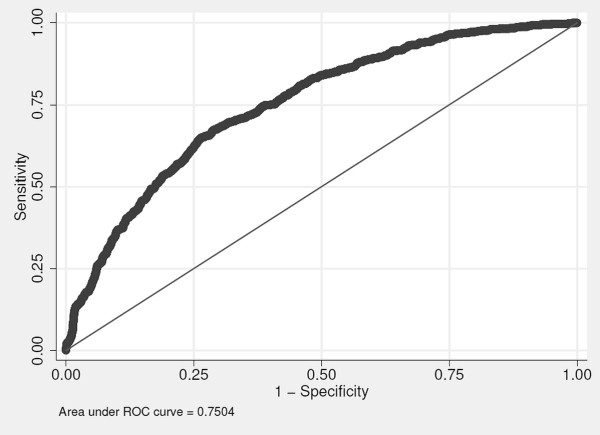
ROC curve for chronic bronchitis using all predictors of interest in the final model.

As an additional set of sensitivity analyses, we considered potential interactions between each predictor of interest with sex and race for each predictor of interest separately and when entered as a group. None of the interaction terms remained significant after adjustment for multiple hypothesis tests. Figure 3 shows the receiver operator curve for all of the aforementioned predictors of interest in the model for chronic bronchitis as the outcome. The area under the curve is 0.75.

## Discussion

We showed in a large cohort of 2,703 COPD subjects that chronic bronchitics were younger, had a reduced 6-minute walk distance, had a greater exacerbation frequency, were more likely to have gastroesophageal reflux, allergic ocular and nasal symptoms, histories of asthma and bronchitis, and were more likely to be current smokers, Caucasian, and male compared to those without CB, thereby validating our prior clinical findings in a smaller cohort that was less well characterized from a radiographic standpoint [2]. These findings were independent of the presence of active smoking. We also showed that the CB + group had a greater segmental wall area percent, Pi10, and Pi15 despite similar degrees of emphysema and gas trapping. Finally, we showed that the variables independently associated with CB were lung function, allergic rhinitis symptoms, history of acute bronchitis and asthma, airway wall thickness, exposure to dusts, gender, race, and current smoking in multivariate analysis. Using our prediction model, one can help identify those with the clinical phenotype of CB that is at higher risk for poor outcomes. To our knowledge, this is the first analysis that combines quantitative CT and clinical variables as predictors of CB in a large cohort of COPD subjects.

CB affects approximately 10 million individuals in the United States, and the majority are between 44 and 65 years of age [16]. CB has been associated with numerous adverse clinical outcomes. Exacerbation frequency has been shown to be greater in patients with COPD and CB in several studies [3,17]. Seemungal et al. found that CB significantly increased the odds of having frequent exacerbations in a group of 70 patients [17]. A cross-sectional analysis of 433 patients also similarly found an increased risk of exacerbation among individuals with CB [3].

CB may increase all-cause mortality as well, independent of the level of airflow obstruction. Some but not all studies have shown CB to be an independent risk factor for death. In a study by Pelkonen et al., the multivariable hazard ratios for all-cause mortality in those with persistent CB was 1.64 (95% CI, 1.23–2.19) after adjustment for lung function [9]. In a post hoc analysis of the National Emphysema Treatment Trial, severe CB, defined as chronic cough, phlegm, and chest trouble, was associated with a higher mortality and hospitalization rate in those treated with medical therapy alone [18]. CB has also been shown to hasten the rate of lung function decline and reduce health related quality of life [2,19,20].

Radiographic characterization of COPD has been performed extensively in the past, [17,18,21] but few studies have related CT parameters to the clinical phenotype of CB. A small study of 42 COPD subjects showed that airway wall thickness was greater in those with CB compared to those without [22]. We have shown that percent emphysema and percent gas trapping were no different between those with CB and those without in 1061 moderate to severe COPD subjects [2]. We validate these prior findings on our study but add quantitative airway measurements in several compartments. Our findings of increased mean segmental wall area percent, Pi10, and Pi15 (“medium sized airways”) in chronic bronchitics is consistent with the notion that the clinical phenomenon of chronic bronchitis arises from mucus hypersecretion from larger more proximal airways, as opposed to small airway disease which is often difficult to detect clinically [11,23]. This is, however, hypothesis generating and would require further study for confirmation. Although the differences in WA% were small, the composite variable of wall thickness was significantly associated with CB.

One of the strongest predictors of CB in this cohort was current smoking. Smoking causes airway inflammation and oxidative stress which induces mucin gene expression [1]. Active smokers have been shown in other studies to be at greater risk of CB. In a 30-year longitudinal study of 1,711 Finnish men, the cumulative incidence of CB was 42% in continuous smokers, 26% in ex-smokers, and 22% in never smokers [9]. A recent metaanalysis found that among 101 studies, ever smoking and current smoking were associated with relative risks of 2.69 and 3.41, respectively, for CB [24]. We showed that current smoking was associated with an odds ratio of 3.53 for CB.

Although the primary risk factor for CB is smoking, it should be noted that CB has been described in up to 22% of never smokers, [9] suggesting that other risk factors may exist. Other potential risk factors include inhalational exposures to biomass fuels, dusts, and chemical fumes [25,26]. A survey of South African adults found occupational exposure to be a risk factor for CB in men (OR 2.6; 95% CI 1.7–4.0) and domestic fuel exposure to be a risk factor for CB in women (OR, 1.9; 95% CI 1.4–2.6) [27]. In a recent analysis of 5,858 smokers or ex-smokers without COPD, patients with CB were more likely to have been exposed to fumes at work (76.4 vs. 60.9%, P < 0.001) or to have worked more than 1 year at a dusty job (76 vs. 57%, P < 0.001) [28]. We found that subjects with CB had a 1.4-fold and 1.3-fold increased risk for exposure to dusts and occupational exposures, respectively. Although occupational exposures were not statistically significant in the multivariate predictive model, we present some evidence of their association in univariate analysis, providing more evidence of a causal association.

Another potential risk factor for CB is the presence of gastroesophageal reflux disease (GERD), possibly by pulmonary aspiration of refluxed gastric contents producing acid-induced injury and infection or neurally mediated reflex bronchoconstriction secondary to irritation of esophageal mucosa [29]. There is increasing evidence that not only is GERD a significant comorbidity but also that it is a risk factor for COPD related outcomes. For example, the Evaluation of COPD Longitudinally to Identify Predictive Surrogate Endpoints (ECLIPSE) study identified GERD as a risk factor for COPD exacerbations in longitudinal follow-up [30]. In the Azithromycin for Prevention of Exacerbations of COPD (MACRO) trial, GERD was associated with an increased risk of COPD exacerbations [31]. In our study, we demonstrated that GERD was more common in those with CB and that GERD conferred a slightly increased odds ratio of having CB in multivariate analysis that almost reached statistical significance.

Other risk factors identified in our study include a history of acute bronchitis, and allergic nasal symptoms. Repeated bouts of acute bronchitis has been considered a risk factor for the development of CB, [32] possibly by the development of persistent inflammation and mucus hypersecretion after multiple episodes of infectious bronchitis. Mucus hypersecretion is a signature pathologic feature of asthma, and asthma’s association with allergic rhinitis is strong. The association between chronic bronchitis and allergic nasal symptoms suggests a link between upper and lower airway inflammation that has commonly been described in asthma. This raises the possibility of a common pathophysiology between CB and asthma, which deserves further attention.

The influence of gender on CB continues to be a matter of debate. Many studies have found that CB affects men more than women, [2,33,34] but in the 2009 National Center for Health Statistics report, 67.8% of patients with CB were women [35]. The reasons for the higher prevalence of CB in women compared with men is unclear, but may be due to hormonal influences, sex differences in symptom reporting, and sex diagnostic bias. Our study showed that men were more likely to be affected by CB and that gender was an independent predictor of CB in multivariate analysis.

Despite the clinical and CT findings in this robust cohort of unselected COPD subjects, there are several limitations that are worthy of mention. First, the assessment of medical history and exposures was by self-report, potentially leading to recall bias. The outcome of interest itself is somewhat subjective as well (chronic cough and phlegm). Although the identified clinical and computed tomographic factors were used in a prediction model, one could argue that some of predictors of interest are caused by chronic bronchitis, not the result of it (eg, airway wall thickness). In addition, it is unclear why the FVC is lower in chronic bronchitics in univariate analysis but higher in multivariate analysis; this phenomenon is most likely due to the confounding factors of differences in FEV_1_, race, and gender. However, the predictors of interest were chosen because of the more likely causal relationship of CB. Nonetheless, our analysis offers a comprehensive characterization of chronic bronchitis in COPD as well as a prediction model for CB using several clinical and computed tomographic parameters.

## Conclusions

In conclusion, we show that allergic rhinitis, acute bronchitis, asthma, male gender, Caucasian race, lung function, current smoking, and airway wall thickness are associated with clinical phenotype of CB. We also show in a large cohort that CB is associated with greater exacerbation frequency, worse health related quality of life, and a lower 6-minute walk distance. Identification of these clinical and CT parameters can help predict the clinical phenotype of CB and can help stratify patients into a group at higher risk for poor outcomes. This group should receive treatment for their comorbidities and receive targeted therapy towards smoking cessation.

## Abbreviations

CB: Chronic bronchitis; CI: Confidence interval; COPD: Chronic obstructive pulmonary disease; CT: Computed tomography; FEV1: Forced expiratory volume in one second; FVC: Forced vital capacity; GERD: Gastroesophageal reflux disease; HRQoL: Health related quality of life; OR: Odds ratio; Pi10: Square root of wall area of 10mm airway; Pi15: Square root of wall area of 15mm airway; RR: Relative risk; WA%: Wall area percent.

## Competing interests

This study was supported by the NHLBI R01 HL089856 and R01 HL08989.

VK is supported by NHLBI K23HL094696-03.

VK has participated in clinical trials sponsored by Boehringer Ingelheim, Glaxo-Smith-Kline, and Roche pharmaceuticals. AD, JC, and CHM report no conflicts. APC has been a consultant for VIDA diagnostics. Also, APC has participated in clinical trials sponsored by Boehringer Ingelheim, Glaxo-Smith-Kline, and Astra-Zeneca, and Forest. MKH has participated in advisory boards for Boehringer Ingelheim, Pfizer, GlaxoSmithKline, Genentech, Novartis, and Medimmune; participated on speaker’s bureaus for Boehringer Ingelheim, Pfizer, GlaxoSmithKline, Forest, Grifols therapeutics, and the National Association for Continuing Education, and WebMD; has consulted for Novartis, Ikaria and United Biosource Corporation; and has received royalties from UpToDate and ePocrates. GW has received grants from the NHLBI to perform quantitative image analysis and have been a paid consultant for MedImmune and Spiration, and his spouse is an employee of Merck Research Laboratories. Over the last three years, BJM has participated in advisory boards, speaker bureaus, consultations and multi-center clinical trials with funding from the National Heart Lung and Blood Institute, Abbott, Astellas, AstraZeneca, Boerhinger-Ingelheim, Coviden, Dey, Forest, GlaxoSmithKline, Merck, MedImmune, NABI, Novartis, Pfizer, Respironics, Sepracor, Sequal and Talecris. EKS received grant support from GlaxoSmithKline for studies of COPD genetics, and he received honoraria and consulting fees from AstraZeneca, Merck, and GlaxoSmithKline. DAL’s institution and laboratory receives research support from the National Heart Lung and Blood Institute, Siemens, Inc, Perceptive Imaging, Inc, and Centocor, Inc, Inc. Dr Lynch is a consultant to Perceptive Imaging, Inc, Boehringer Ingelheim, Inc, Genentech, Inc, Gilead, Inc, Veracyte, Inc and Intermune, Inc. GJC has served on Advisory Committees for Boehringer Ingelheim, CSA, Amirall and Holaira. All of these sums are less than $2,500. GJC has received research grants from: Boehringer Ingelheim, AstraZeneca, MedImmune, Pearl, Actelion, Glaxo-Smith-Kline, Forest, Aeris, Therapeutics, Pulmonx and PneumRx. All research grant monies are deposited and controlled by Temple University.

## Authors’ contributions

All authors participated in the study design, data analysis, and writing of the manuscript. VK is the guarantor for the overall content.
